# Assessment of the Theranostic Potential of Gold Nanostars—A Multimodal Imaging and Photothermal Treatment Study

**DOI:** 10.3390/nano10112112

**Published:** 2020-10-23

**Authors:** Antoine D’Hollander, Greetje Vande Velde, Hilde Jans, Bram Vanspauwen, Elien Vermeersch, Jithin Jose, Tom Struys, Tim Stakenborg, Liesbet Lagae, Uwe Himmelreich

**Affiliations:** 1 Biomedical MRI, Department of Imaging and Pathology, Faculty of Medicine, KU Leuven, Herestraat 49, 3000 Leuven, Belgium; antoinedhollander@hotmail.com (A.D.); greetje.vandevelde@kuleuven.be (G.V.V.); elien.vermeersch@kuleuven.be (E.V.); 2 Molecular Small Animal Imaging Center (MoSAIC), KU Leuven, Herestraat 49, 3000 Leuven, Belgium; 3 Department of Life Science Technology, IMEC, Kapeldreef 75, 3001 Leuven, Belgium; Hilde.Jans@imec.be (H.J.); brmvanspauwen@gmail.com (B.V.); Tim.Stakenborg@imec.be (T.S.); liesbet.lagae@imec.be (L.L.); 4 Fujifilm Visualsonics, Joop Geesinkweg140, 1114 AB Amsterdam, The Netherlands; jithin.jose@fujifilm.com; 5 Lab of Histology, Biomedical Research Institute, Hasselt University, Agora Laan Gebouw C, 3590 Diepenbeek, Belgium; tom.struys@sprofit.com; 6 Department of Physics, Faculty of Sciences, Laboratory of Soft Matter and Biophysics, KU Leuven, Celestijnenlaan 200D, 3001 Leuven, Belgium

**Keywords:** gold nanostars, photothermal therapy, photoacoustic imaging, computed tomography, nanoparticles, cancer, theranostic agents

## Abstract

Gold nanoparticles offer the possibility to combine both imaging and therapy of otherwise difficult to treat tumors. To validate and further improve their potential, we describe the use of gold nanostars that were functionalized with a polyethyleneglycol-maleimide coating for in vitro and in vivo photoacoustic imaging (PAI), computed tomography (CT), as well as photothermal therapy (PTT) of cancer cells and tumor masses, respectively. Nanostar shaped particles show a high absorption coefficient in the near infrared region and have a hydrodynamic size in biological medium around 100 nm, which allows optimal intra-tumoral retention. Using these nanostars for in vitro labeling of tumor cells, high intracellular nanostar concentrations could be achieved, resulting in high PAI and CT contrast and effective PTT. By injecting the nanostars intratumorally, high contrast could be generated in vivo using PAI and CT, which allowed successful multi-modal tumor imaging. PTT was successfully induced, resulting in tumor cell death and subsequent inhibition of tumor growth. Therefore, gold nanostars are versatile theranostic agents for tumor therapy.

## 1. Introduction

‘Nanotheranostics’—referring to the use of nanotechnology for combined imaging and treatment of diseases—is currently an active research field as combining diagnosis with therapy has several advantages. The knowledge of the biodistribution of therapeutic agents through imaging can improve the guidance and initiation of cancer therapy. This can, for example, help to decide on the best time point for applying photothermal therapy. In addition, therapy success can be assessed at earlier time points and follow-up of therapy efficiency can be improved drastically. In the clinic, this will ultimately result in earlier intervention, better patient management, and improved prognosis [1]. Within the field of nanotheranostics, especially gold nanoparticles (AuNPs) that show a localized surface plasmon resonance (LSPR) can have a significant impact, as the LSPR effect can be used for both imaging and therapy [2]. The LSPR effect can be explained by the collective oscillation of the conduction band electrons due to light. This collective oscillation induces an enhanced absorption and scattering of the light [3]. The scattering properties of the light can be exploited by several imaging techniques including surface enhanced Raman scattering [4,5] and darkfield microscopy [2]. The light energy that is not scattered by the particles but absorbed, is converted into heat. This specific heat generation is the basis for both photoacoustic imaging (PAI) and photothermal therapy (PTT) [6,7].

PAI is based on the acoustic waves generated by the thermo-elastic expansion that occurs when a specific compound absorbs a pulsed electromagnetic wave [8]. Exogenous contrast (e.g., carbon nanotubes, AuNPs) have shown to generate higher PAI contrast compared to endogenous molecules (e.g., hemoglobin) [9]. Due to the negligible scattering of ultrasound in tissue compared to light, a relatively high imaging depth of approximately 5 cm is possible with PAI [10]. PTT on the other hand is also based on the heat conversion of AuNPs during irradiation with a continuous laser [11]. Since cancer cells are more sensitive to heat than other cell types, a temperature increase above 43 °C is lethal due to the inability to remove the heat in poorly vascularized tumor tissue [12].

For PAI and PTT, the ratio between the light scattering and absorption properties per AuNP is crucial. Hereto, several shapes of AuNPs have been studied ranging from nanorods, nanoshells, nanocages to nanostars [8]. Usually, nanostars have higher absorption vs. scattering coefficients compared to nanorods and nanoshells, but similar to nanocages [11,13]. This higher absorption coefficient is the crucial parameter for efficient heat conversion, important for PAI and PTT. Several studies have examined the use of gold nanorods as a contrast agent for PAI in tumors [14,15,16,17,18]. For nanostar-shaped AuNPs, mainly non-quantitative in vitro data [19] and in vivo mapping of the lymphatic system and first results on tumor imaging have been shown [20,21]. We have recently demonstrated their tumor-targeting ability using PAI [22]. For PTT of tumors, several groups have reported on nanostars as an effective in vitro PTT agent [13,19,21,23,24], but many questions remain regarding the PTT efficiency using these nanostars in vivo [23,25].

Alternatively, AuNPs can also be imaged with computed tomography (CT), since they absorb X-rays more efficiently compared to frequently used contrast agents such as iodine-based compounds [26,27]. In general, heavy atoms are frequently used as a contrast agent for X-ray based CT for diagnostic imaging in the clinic and for preclinical research [26]. Nonetheless, relatively high local AuNP concentrations are needed to generate sufficient contrast for CT, in particular for small voxel sizes as required for preclinical imaging applications [28]. AuNPs, such as nanorods or spheres, have shown their effectiveness as blood-pool and tumor-targeting contrast agent using CT [26,29]. However, for nanostars, quantitative in vitro and in vivo studies regarding their potential as contrast agents for CT are lacking [19].

Several routes have been explored to administer AuNPs as a theranostic agent against cancer, but many questions remain. Intratumoral administration is the most straight forward way for theranostic application, while i.v. injection has shown mixed results in terms of tumor accumulation [30,31]. Few reports showed reasonable tumor accumulation where the charge of the nanoparticles played a crucial role for tumor targeting [26,32]. Even functionalizing the nanoparticles with biological ligands gave different outcomes in terms of intra-tumor accumulation [33,34]. Active targeting, suggested to overcome problems with low intra-tumor accumulation after intravenous delivery, may have no influence on tumor uptake but does on the distribution within the tumor [33]. To improve biocompatibility and active targeting, AuNPs are frequently coated through an Au-S-bond. These non-covalent bonds are subject to potential thermal instability, releasing part of the coating material [35].

AuNPs have to fulfill some essential requirements to be applicable for in vivo theranostic photothermal approaches. First, the absorption band of the AuNP must be tuned to the near infrared region (NIR) frequency range for having maximal contrast generation and therapy efficiency due to its relative high depth penetration [36]. Second, the diameter of the AuNPs has to be around or below 100 nm to cross leaky blood vessels and being retained in the tumor. Third, charged particles are favored since such particles show better retention after intra-tumor injection because of immediate interaction with the tumor cells [37].

Exploiting the advantage of the specific high absorption capacity of nanostars, we have studied the potential of nanostars for PAI/CT and PTT in vitro and in vivo. In this study, we first optimized the synthesis and functionalization of nanostars for efficient uptake by tumor cells and assessed their PAI and PTT capabilities in vitro using an ovarian cancer cell line (SKOV3). For in vivo validation, gold nanostars were intratumorally injected in a xenograft mouse model and their local distribution in the tumor assessed with CT and PAI. Finally, photothermal therapy was performed and evaluated using bioluminescence imaging (BLI), magnetic resonance imaging (MRI), and histology.

## 2. Materials and Methods

### 2.1. Synthesis and Chemical Functionalization of Nanostars

Gold nanostars were prepared based on the procedure described by Hao et al. [38] and further optimized by Van de Broek et al. [39]. In brief, 2 mg bis(psulfonatophenyl) phenylphosphine dihydrate dipotassium (BSPP; Strem Chemicals, Newburyport, MA, USA) and 100 μL H_2_O_2_ (30% *v/v*, Air Products, Vilvoorde, Belgium) were added to 50 mL of a 6.8 × 10^−3^ M aqueous sodium citrate solution (Acros Organics, Geel, Belgium). In a next step, 100 μL of 0.075 M HAuCl_4_ (Acros Organics) was added slowly under constant stirring at room temperature. By using an Atlas Syringe Pump (Syrris, Ruisbroek, Belgium) a slower addition rate of 12.5 µL/min was used in comparison to previously published articles in order to achieve the desired shape and size [36]. The 50 mL AuNP suspension was centrifuged at 4500 rpm for 1 h and the pellet was re-suspended in 10 mL of water. The star-shape of the AuNPs was stabilized using a disulfide molecule, according to Lin et al. [40]. Hereby, 1 mL of an 1.2 mM disulfide (S-(CH_2_)_11_-(O-CH_2_-CH_2_)_6_-O-CH_2_-CO-NH-(CH_2_)_2_-maleimide)_2_ (Prochimia, Sopot, Poland) solution was added to 10 mL of the AuNP suspension mixed with 100 μL 0.5 M NaOH (Merck, Overijse, Belgium). After 90 min of shaking, the mixture was centrifuged at 4000 rpm for 60 min and re-suspended in water resulting in an optical density of ~1 at their maximum plasmon band. These nanostars were characterized in water and cell culture medium using UV-Vis absorption spectroscopy (Shimadzu UV-1601PC, Brussels, Belgium), dynamic light scattering (DLS; Malvern Nanosizer, WR, United Kingdom) and transmission electron microscopy (TEM; Tecnai F30, FEI company, Eindhoven, The Netherlands). A terminal maleimide group was chosen for future functionalization with anti/nanobodies and targeting of specific cell types as described [22].

### 2.2. Dynamic Light Scattering and Zeta Protocol

For dynamic light scattering (DLS), the hydrodynamic diameters of the nanostars under investigation were measured using a Zetasizer Nano ZS90 DLS system equipped with a red (633 nm) laser and an Avalanche photodiode detector (APD) (quantum efficiency > 50% at 633 nm) (Malvern Instruments Ltd., Malvern, UK). Hydrodynamic diameters represent estimates of average diameters as the Zetasizer Nano ZS90 DLS system is optimized for spherical nanoparticles and not nanostars. A 1.5 mL semi-micro cuvette was used as sample container. The ‘DTS applications 5.10′ software was used to analyze the data. All sizes reported here were based on intensity average where the intensity was observed using a non-negative least squares (NNLS) analysis method. For each sample, two DLS measurements were conducted with a fixed 15 runs and each run lasts 10 s. A detection angle of 173° was chosen for the size measurement.

For determining the zeta potential, an average was taken on three distinct measurements where the nanostars were dissolved in water. A u-shaped polycarbonate flow cell with embedded electrodes at either end, referred to as ‘clear disposable zeta cell’, was used during these measurements. As with the size experiments the ‘DTS applications 5.10′ software was used to process the data.

### 2.3. Transmission Electron Microscopy

We used a transmission electron microscopy (TEM) protocol as previously described [41]. In more detail, tumor tissue was collected after sacrificing the animals. Tissue was cut into cubes of 2 mm^3^ and fixed overnight in 2% glutaraldehyde and 0.05 M sodium cacodylate buffer (pH 7.3) at 4 °C. Tissue samples were post-fixed in 2% OsO_4_ in 0.05 M sodium cacodylate buffer (pH 7.3) for 1 h and stained with 2% uranyl acetate in 10% acetone for 20 min. Next, samples were dehydrated in graded concentrations of acetone and were embedded in epoxyresin (Araldite). Semi-thin slices (500 nm) were cut, stained with toluidine-blue and used for selecting regions of interest. Ultra-thin sections were mounted on 0.7% formvar coated grids, contrasted with uranyl acetate followed by lead citrate and examined with a Philips EM 208 transmission electron microscope operated at 80 kV. Digital images were taken with the MORADA 10/12 camera (Olympus, Hamburg, Germany). TEM analysis was performed with a Philips EM 208 S electron microscope (Philips, Eindhoven, The Netherlands). The microscope was provided with a Morada Soft Imaging System camera to acquire high resolution images of the evaluated samples. The images were processed digitally with the iTEM-FEI software (Olympus SIS, Münster, Germany).

### 2.4. Cell Culture

SKOV3 cells (ATCC^®^ HTB77, Cedex, France) were cultured in Roswell Park Memorial Institute medium (RPMI) 1640 medium supplemented with 10% fetal calf serum, 50 units/L penicillin, 50 µg/mL streptomycin, and 2 mM L-glutamine. Cells were incubated at 37 °C in a 5% CO_2_ environment. All cell culture reagents were obtained from Life Technologies (Gent, Belgium). The SKOV3 cells were transduced with a lentiviral vector (LV-CMV-eGFP-T2A-fLuc) to stably express eGFP and firefly luciferase [42].

### 2.5. Inductively Coupled Plasma Optical Emission Spectroscopy (ICP-OES)

For uptake confirmation, 100,000 cells per well were seeded in a 12-well plate. After 24 h, nanostars (2.3 × 10^10^ particles in 1 mL) were added to the cells and incubation continued for different time periods (1, 3, 6, 12 and 24 h). Next, cells were washed with PBS and again incubated overnight with fresh medium. After trypsinization, 100,000 cells were acid-digested with Kingswater (HCl/HNO_3_ with a ratio of 3:1) and diluted with de-ionized water to a volume of 10 mL for inductively coupled plasma optical emission spectroscopy (ICP-OES) (3300 DV, Perkin-Elmer, Waltham, MA, USA). Reference standards were prepared by dissolving HAuCl_4_ to final concentrations between 0.1 and 2 ppm.

### 2.6. In Vivo Xenograft Model and Nanostar Administration

Female Hsd:Athymic *Nude-Foxn1^nu^* mice were used (8 weeks, Harlan, Horst, The Netherlands) during these experiments. All animal experiments were approved by the local animal ethics committee of the KU Leuven and were performed according to the national and European regulations. Animals were kept in individually ventilated cages with food and water ad libitum. A total number of 1 × 10^7^ SKOV3 tumor cells suspended in 100 µL were injected into each hind limb of the mice and left for two weeks to grow into solid tumors [22].

After formation of tumors (size > 200 µm^3^), 100 µL containing 9.2 × 10^11^ NPs/mL were injected into the tumor on the left hind limb. For controls, 100 µL PBS was injected into the right tumor in all animals (sham control). During all imaging experiments, tumor cell and nanostar injections, the animals were anesthetized with 1.5% isoflurane in 100% O_2_. The body temperature and respiration rate were monitored and maintained at 37 °C and 80–120 min^−1^, respectively. For the imaging experiments, three mice were used per condition while for the therapy experiments, six animals were used per condition.

### 2.7. Photoacoustic Imaging (PAI)

PAI was performed with a Vevo^®^ Lazer 2000 (Fujifilms Visualsonics, Amsterdam, The Netherlands) using a 10 ns pulse laser (680–900 nm) with an energy fluence of 20 mJ/cm^2^ and 21 MHz central frequency. These conditions were maintained for the in vitro and in vivo experiments. For in vitro PAI, 200,000 cells were suspended in 100 μL PBS and mixed with 100 μL warm agar (Sigma, Diegem, Belgium). This solution was added and solidified in a bigger agar block. During in vivo PAI, ultrasound imaging is used to determine the tumor location, after which PAI was performed to validate nanostar injection.

### 2.8. Computed Tomography (CT)

In vivo and in vitro CT images were acquired using an in vivo microCT scanner (Skyscan 1076, Bruker microCT, Kontich, Belgium) with the following settings: 50 kV X-ray source, 200 µA source current, 0.5 mm Al filter, 120 ms exposure time, 22 × 29 mm field of view, 0.7° rotation step over a total angel of 180°, which results in a tomographic dataset with a 35 µm isotropic resolution. The data has been processed using software from the manufacturer (NRecon, Dataviewer, CTvox and CTan). Sample preparation was similar for CT as for PAI.

### 2.9. Photothermal Therapy (PTT)

In vitro and in vivo PTT experiments were performed using a home-built laser setup [12]. For in vitro PTT, nanostars (2.3 × 10^10^ particles in 1 mL) were added to a 12-well plate containing 100,000 cells/well that were pre-incubated overnight. Nanostar concentrations were calculated as previously described in the supplementary methods section of [13]. The cells were then incubated with the nanostars for 24 h or with fresh medium as a control sample and washed twice with PBS. Next, the incubated cells were exposed to laser irradiation (690 nm, 20 W/cm^2^, 5 min) and incubated for an additional 2 h at 37 °C. Afterwards, the cells were washed with PBS. The cell viability was assessed using a live/dead staining (Calcein AM/Hoechst; Life Technologies Europe B.V., Gent, Belgium). After staining, the cells were imaged using a fluorescence microscope with 5× objective (CellR system, Olympus, Aartselaar, Belgium). During the in vivo experiments, the mice were irradiated for five minutes with a laser (λ = 690 nm; 2 W/cm^2^), inducing PTT one day after nanostar administration. During this laser treatment, the mice were anesthetized with ketamine/domitor [43].

### 2.10. Magnetic Resonance Imaging (MRI)

MRI data acquisition and processing was similar to previous reports [44]. In brief, in vivo MRI was performed using a 9.4 T small animal MRI system (BioSpec, Bruker Biospin, Ettlingen, Germany) equipped with a gradient insert with a maximum gradient strength of 600 mT m^−1^. A quadrature transmit-receive coil (Bruker Biospin) with an inner diameter of 7 cm was used for data acquisition. The MRI protocol included a spin echo sequence with TR = 6000 ms, TE = 15.8 ms, matrix = 200 × 200 mm, field of view 40 × 40 mm, slice thickness = 0.5 µm and 40 slices.

### 2.11. Bioluminescence Imaging (BLI)

BLI experiments were performed with an IVIS 100 imaging system (Perkin Elmer, Massachusetts, United States). The mice were injected intra-venously (i.v.) with D-luciferin (126 mg/kg body weight, Promega) dissolved in PBS (15 mg/kg). Afterwards, they were placed in the IVIS 100 imaging system and one image frame per second was acquired until a signal intensity plateau was reached. The following settings were used: 1 s exposure time, FOV of 10 cm, binning of 4, and an f/stop of 8. For in vivo quantification of fLuc reporter gene activity, the data were analyzed as photon flux per second (p/s) from a 1 cm^2^ circular ROI located on the tumor using the Living Image software (version 2.50.1, Perkin Elmer). The signal intensity values at different time points (day 0, 1, 5, 8 and 15) were presented relative to the BLI signal of the same mouse at day 0.

### 2.12. Histopathology

Mice were sacrificed and transcardially perfused with 4% paraformaldehyde (PFA) in PBS 15 days after treatment. The tumors were dissected and post fixed overnight in 4% PFA. The tumor tissue was embedded in paraffin and sectioned into 5 μm slices, stained with hematoxylin and eosin (H&E) and visualized with a Mirax desk scanner (Zeiss, Jena, Germany). In addition to histopathology, TEM analysis was performed on the tumor tissue following the protocol supplied in the supplementary information of Trekker et al. [45].

### 2.13. Data Analysis

For quantification of CT and PAI data, contrast to noise ratios (CNR = (SI_0_ − SI_1_/σ(noise)) were calculated. Statistical analyses were executed on the quantitative data using a paired *t*-test where the degree of significance is indicated with * *p* < 0.05; ** *p* < 0.01; *** *p* < 0.001.

## 3. Results

### 3.1. Synthesis and Functionalization of Nanostar-Shaped AuNPs Optimized for In Vivo Use

**Figure 1:**
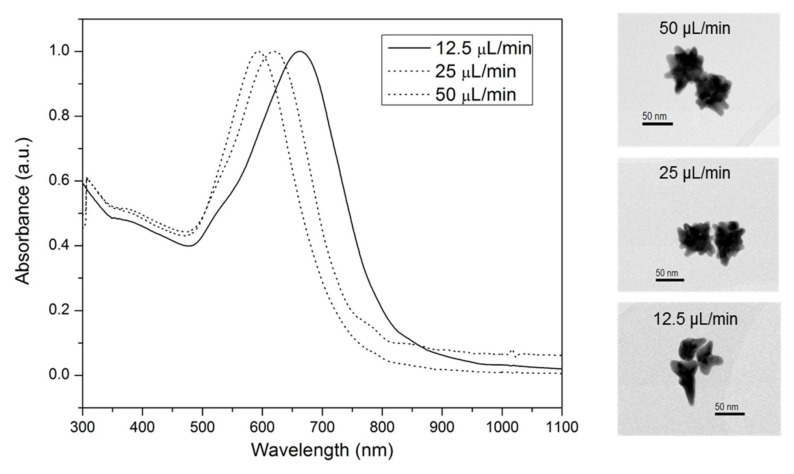
UV-Vis absorption spectroscopy of nanostars using different flow rates for the gold salt. Note the shift to the near infrared region (NIR) region with lower flow rates. TEM images of the nanostars that were generated with flow rates of 50 μL/min, 25 μL/min and 12.5 μL/min (from top to bottom). These images suggest that the amount and length of spikes change with different flow rates. The diameter of the nanostars did not significantly change as confirmed by dynamic light scattering (DLS) where no significant increase was noticed with lower flow rates (50 μL/min: 66.0 ± 1.5 nm; 25 μL/min: 67.5 ± 2.7 nm: 12.5 μL/min: 66.3 ± 7.8 nm; data not shown).

To be able to use nanostars as a theranostic agent in vivo, they need to be stable under physiological conditions, preferably absorb the light in the NIR-range and have a size around 100 nm for optimal retention in tumors. To meet these requirements, we optimized a two-step method by changing the flow rate of the HAuCl_4_ to 12.5 μL/min during synthesis and by functionalizing them with a self-assembled monolayer to stabilize their specific shape (Figure 1) [13]. The resulting nanostars showed an improved plasmon absorption band around 670 nm and 679 nm (Supplementary Figure S1). This red shift indicated a successful chemical functionalization with the disulfide, resulting from a local refractive index change due to the chemisorption of the disulfide onto the nanostars. These results were confirmed by DLS, showing an average diameter of 66.3 ± 7.8 nm for the synthesized nanostars and 75.0 nm ± 5.6 nm for the further functionalized nanostars. After functionalization with disulfide-PEG-maleimide, the zeta potential was −41.3 ± 1.2 mV, indicating negatively charged nanostars (Supplementary Figure S2). The branched shape of the nanostars was confirmed using TEM (Figure 1). Incubating the nanostars in biological medium for one week at 37 °C did not show any indication of instability, confirmed by the absence of peak broadening of the LSPR band or increase in diameter of the nanostars over time (Supplementary Figure S2). Still, an increase of the diameter to 100.6 ± 1.8 nm was seen immediately after incubation in cell culture medium due to the formation of a protein corona. As a consequence, these optimized nanostars are suitable for in vitro and in vivo theranostic applications.

**Figure 2:**
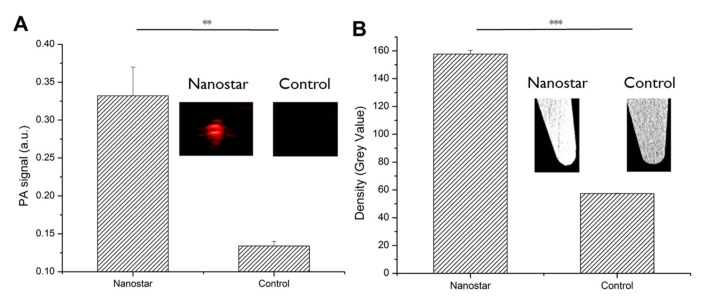
(**A**) Photoacoustic images of nanostars and water in tubes, which were quantified by plotting the signal amplitudes. (**B**) Computed tomography (CT) images of microcentrifuge tubes either filled with water or nanostars suspension. The corresponding signal amplitudes were used for quantification. (Error bars represent SD of triplicate samples; * *p* < 0.05, ** *p* < 0.01, *** *p* < 0.001).

After functionalization, the capability of the nanostars to generate image contrast was studied in water using a concentration of 1.55 mg Au/mL. A PAI signal of 0.33 ± 0.04 a.u. was measured for the nanostars while water shows a signal of 0.13 ± 0.01 a.u. (Figure 2). For CT, a signal of 175.67 ± 2.74 and 57.33 ± 0.17 for the nanostars and water was measured, respectively. As a consequence, both imaging modalities showed almost an identical CNR of 32.85 for PAI and 32.53 for CT, respectively.

### 3.2. PAI and CT Confirm Efficient Nanostar Uptake by Tumor Cells

In order to test whether tumor cells efficiently take up the nanostars, SKOV3 cells were incubated with these NPs for different time periods (1, 3, 6, 12, and 24 h). Their uptake was examined by using ICP-OES, CT and PAI. ICP-OES measurements showed for the first incubation time points an almost linear increase of intracellular gold concentration while over time this seemed to change into a plateau phase as visualized by the fitting curve in Supplementary Figure S3. After 24 h, an intracellular gold amount of 11.28 ± 0.82 pg Au/cell was observed. TEM confirmed intracellular uptake of the nanostars as they were found inside vesicular structures of the cells but not at the cell membrane.

**Figure 3:**
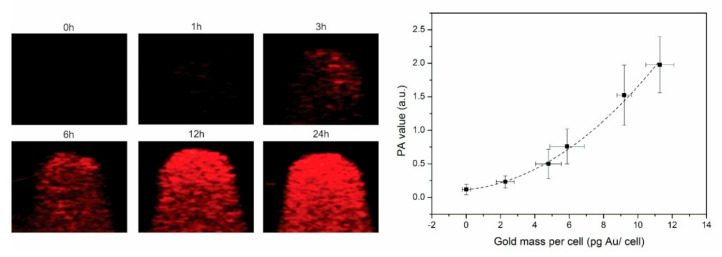
**Left**: PA images of a phantom loaded with tumor cells (n = 3) incubated for different time slots with the nanostars. **Right**: PA signal plotted for the different gold masses per cell, where the dotted line suggests an exponential relation between those 2 variables.

Furthermore, we evaluated whether this uptake was sufficiently high to be visualized by PAI and CT. Hereby, the tumor cells labeled with different amounts of gold nanostars were homogenously suspended in an agar phantom at 1000 cells/μL. PA images shown in Figure 3 indicated an increased PA signal (red pixels) over incubation time, where an exponential correlation was found between the PA signal and intracellular gold concentration. After 24 h, a PAI signal 1.93 ± 0.42 a.u. was measured compared to a PA signal of 0.19 ± 0.02 a.u. for the unlabeled cells. This successful nanostar-labeling resulted in an CNR of 180.73 for PAI and a limit of detection (LOD) of 3.5 pg Au/cell (17 μM).

**Figure 4:**
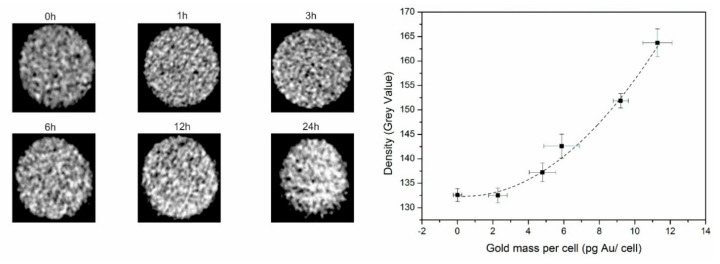
**Left**: CT images of an agar phantom loaded with tumor cells (n = 3) incubated for different time points with the nanostars. **Right**: CT signal quantified as density (grey values) plotted for the different gold masses per cell, where the dotted line suggests an exponential relation between those 2 variables.

For CT, the contrast visualized on a grey intensity scale is shown in Figure 4 where an increase of density was noticed over time. When plotting these density values over the different intracellular gold concentrations, an exponential fit was deducted. After 24 h, a CT signal of 161.33 ± 1.42 was measured for cells labeled for 24 h with nanostars compared to 130.28 ± 3.25 for unlabeled cells. The CT imaging capabilities results in a CNR of 7.36 for CT and a LOD of 5.5 pg Au/cell (28 μM).

### 3.3. Effective In Vitro Photothermal Tumor Cell Ablation Using Gold Nanostars

Since tumor cells were effectively labeled with nanostars, we investigated if the in vitro uptake was sufficient to eradicate tumor cells by PTT. First, the potential of nanostars for heat generation necessary for PTT was investigated using a glass capillary filled with either 4.6 × 10^10^ nanostars/mL or water as a negative control. When irradiating the sample with a 690 nm laser (7 W/cm^2^), a temperature increase of 25 °C was observed for the nanostar suspension, while no temperature increase was measured for the water filled capillary (Supplementary Figure S4).

**Figure 5:**
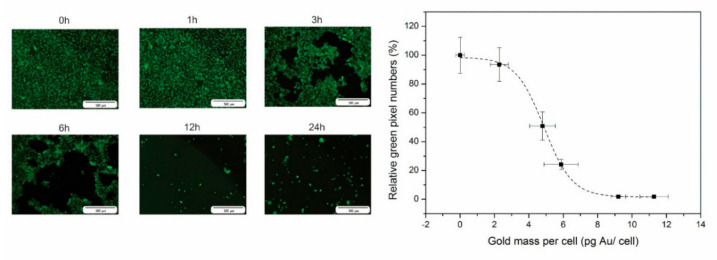
**Left**: Fluorescence images of the living cells after photothermal therapy (PTT) using calcein AM staining for tumor cells (n = 3) incubated for different time periods with the nanostars. **Right**: Relative green pixel numbers plotted against the gold mass per cell. Fluorescence signal intensity is expressed relative to the unlabeled control cells (0 h).

A crucial parameter for determining the effectiveness of PTT is to calculate the cell killing capacity (IC50 value). To determine this value, the tumor cells were labeled with the nanostars using the same conditions as for the imaging experiments. After PTT, cell death was visualized by calcein AM living cells staining and quantified by calculating the total green pixels. As visualized by fluorescence microscopy a radius increase of non-viable cells at the laser spot indicates a higher PTT effectiveness with an increasing intracellular gold concentration (Figure 5). By plotting the relative green fluorescence signal compared to the fluorescence signal of the control cells, a sigmoidal curve was fitted where an IC50 of 4.8 pg Au/cell (23 μM) was calculated.

### 3.4. In Vivo CT and PAI Confirms Nanostar Delivery into Tumors

**Figure 6:**
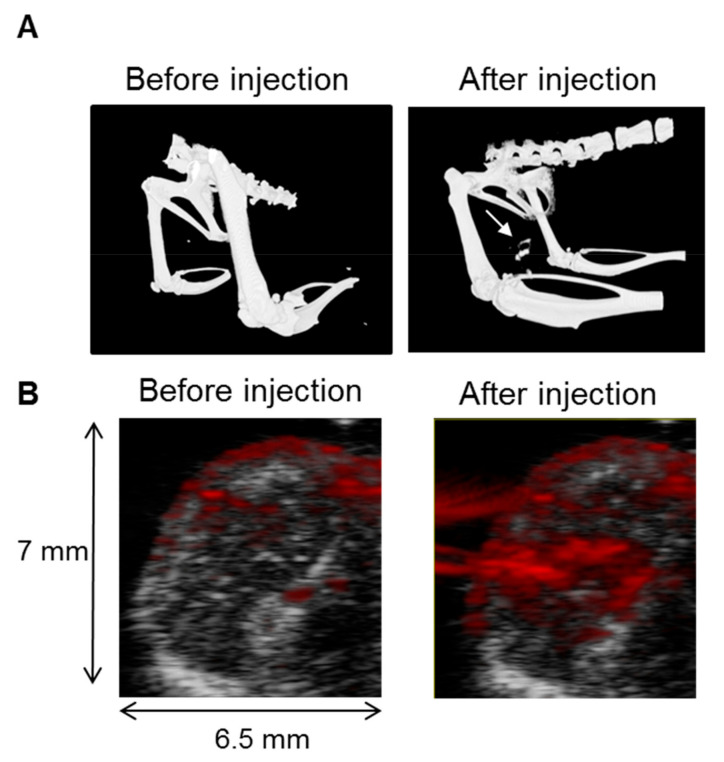
(**A**) CT images before and after injection of gold nanostars into the tumor. An increased contrast was noticed at the tumor site after gold nanostars injection as indicated by the arrow; (**B**) In vivo PA images before and 24 h after nanostar injection. The photoacoustic imaging (PAI) signal (red pixels) is overlaid over the anatomical ultrasound images (grey pixels).

We evaluated if nanostars injected into tumors can be visualized by in vivo imaging. Tumor-bearing mice (n = 3) were imaged with CT and PAI before and one day after gold nanostar injection into the tumor. Before injection, only the contrast between the skeleton and the soft tissue was visible using CT (Figure 6A). After injection of the nanostars, an intense contrast of 94.63 ± 6.77 was observed at the left side of the tumor, corresponding to the place of the tumor (Figure 6A, right). No significant change in contrast was generated in the PBS-injected control tumor with a signal of 74.11 ± 1.43 (Figure 6A, left). A CNR increase of 25.31 was calculated after intratumoral injection of gold nanostars, indicating that intratumoral delivery of nanostars can be monitored with CT in vivo.

Alternatively, we evaluated whether intratumoral nanostar delivery can also be followed up with PAI. Hereby, both ultrasound and photoacoustic images were overlaid as shown in Figure 6B. The ultrasound image was used for localizing and visualizing the tumor. Although some background photoacoustic contrast due to hemoglobin is present before nanostar injection, PAI could be used to visualize the nanostars as indicated by a significant increase in signal intensity compared to the background (1.20 ± 0.17 a.u. compared to the control 0.44 ± 0.05 a.u.) with a CNR of 80.31.

### 3.5. Gold Nanostars Mediate In Vivo Photothermal Therapy

**Figure 7:**
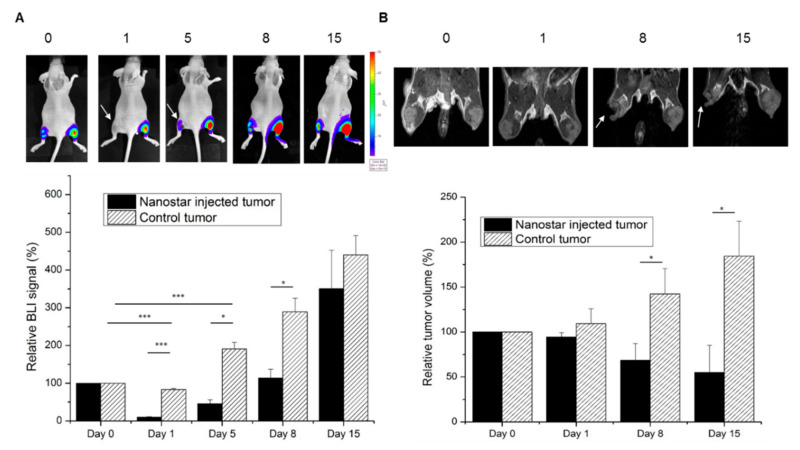
(**A**) In vivo bioluminescence imaging (BLI) before (day 0) and after PTT (days 1, 5, 8) illustrated in a color-coded intensity map. A quantification of the BLI signal intensity relative to day 0 (set to 100% for each mouse) is plotted for both nanostar-injected and control (PBS-injected) tumors (RT); (**B**) MR images of the tumor-bearing hind limbs taken at corresponding time points (days) after nanostar- or PBS-injection. The white arrow indicates the tumor damage (hypointense area) after PTT at the nanostar-injected tumor. The relative mass volumes were quantified relative to day 0 (set at 100% per mice). For both BLI and magnetic resonance imaging (MRI) graphs, the error bars represent SD; n = 6; * *p* < 0.05, ** *p* < 0.01, *** *p* < 0.001.

The effectiveness of nanostars to mediate PTT was studied in a xenograft mouse model (n = 6). The left tumor was used for injection with gold nanostars, while the right tumor acted as control after sham injection with PBS. 24 h after nanostar injection, both tumors were irradiated with a continuous laser (5 min, 690 nm and 2 W/cm^2^). Therapy efficacy was assessed in vivo by using MRI and BLI to evaluate tumor regression and viability. For quantification of the tumor cell viability, the BLI signal was monitored one day before and 0, 1, 5, 8 and 15 days after PTT. The day before PTT and after imaging, nanostars/PBS were injected into the tumors. A decrease in BLI signal intensity was detected for the nanostar-injected tumor at day 0, which was not observed for the PBS injected tumor (Supplementary Figure S5). This is explained by the nanostars absorbing/scattering a part of the BLI signal. This time point was used as baseline for assessment of PTT. One day after PTT, a significant decrease in relative BLI signal intensity to 10.13 ± 1.41% was observed for the nanostar-injected tumor compared to the tumor at day 0, indicating a decrease in viable tumor cells or vessel patency (Figure 7). After 5 days, an increase of the BLI signal intensity was noticed to a relative value of 45.78 ± 10.02%, which indicates partial re-growth of the PTT treated tumor as probably not all tumor cells were photothermally ablated. Although, a significant difference in signal intensity was maintained when compared to the tumor at baseline. In contrast, the right control tumor showed a constant increase in BLI signal intensity over time until a relative BLI signal of 440.24 ± 51.14% after 15 days, confirming that the viability of the control tumor was not affected by laser irradiation. When comparing the nanostar-injected with the control tumor, a significant difference in BLI signal intensity was detected at day 1, 5, and 8, indicating a significant inhibition of tumor growth by PTT.

For providing more information on the therapeutic effect, MR images were acquired on day 0, 1, 8 and 15 to monitor tumor size, anatomy, and heterogeneity. After 8 and 15 days, a relative tumor volume of 68.64 ± 18.45% and 55.25 ± 30.1% was observed for the left tumor compared to day 0, indicating effective PTT (Figure 7B). This was confirmed by the significant difference between this volume and the volume of the control tumor (PBS injection), where a relative tumor volume of 142.27 ± 28.49% at day 5 and 184.12 ± 39.13% at day 8 was measured, respectively. MRI also indicate that the nanostar-injected tumors were not affected evenly by PTT over the whole tumor volume, indicating local differences due to inhomogeneous distribution of the nanostars (Figure 7B).

**Figure 8:**
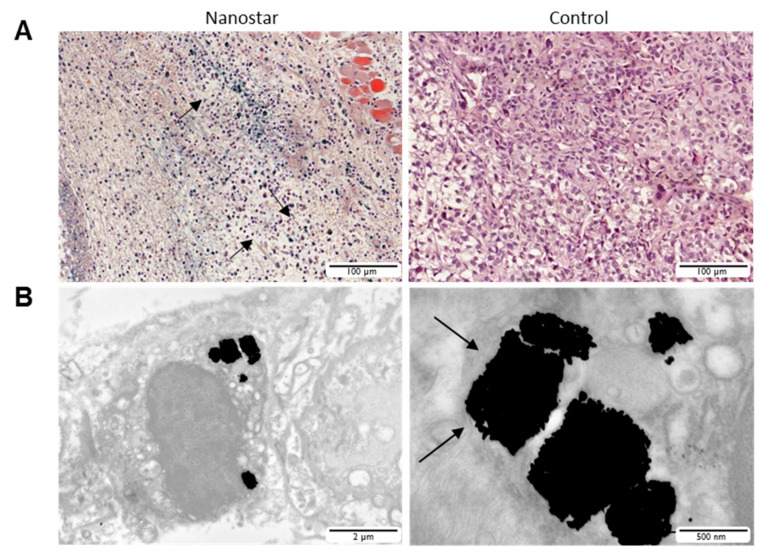
(**A**) Bright field microscopy images of control (right) and nanostar (left) injected hematoxylin and eosin (H&E) stained tumor sections. Defragmented nuclei of the tumor cells could be visualized after therapy. (**B**) Ex vivo TEM images of tumor cells that indicate the presence of NP clusters in endosomes. The right panel shows a zoomed section of these endosomes with vesicular structures visible.

For validation, mice (n = 3) were sacrificed after the first (day 1) and last (day 15) imaging time point following PTT. Tumors were resected and histologically examined. After one day, a distinct blue color was seen on these histological slices originating from the nanostars with an LSPR band of 679 nm, which were visible in the tumor. These nanostars were only noticed in the tumor region where also necrotic cells were visible (Supplementary Figure S6). As can be seen on the H&E stained images, the nuclei were either defragmented or darkened in comparison to the healthy cells (Figure 8). For the PBS-injected control tumors, the blue color due to the nanostars was absent and the majority of cells appeared healthy. At the last imaging time point, no necrotic cells were visible on the histology sections for both the PBS- and nanostar-injected tumors (Supplementary Figure S6).

Using TEM, the presence of nanostar clusters inside vesicles of the cells was confirmed (Figure 8). At the ultrastructural level, tumor cells were identified based on their pleomorphic aspects, increased nuclear/cytoplasmic ratio and distinct anaplasia. The latter was typically represented by nuclear hyperchromatism, thereby confirming that the tumor cells took up the nanostars. These nanostars were present in the cytoplasm and typically stored in membrane-bound compartments (endosomes, arrows in Figure 8B). As the endosomes were packed with nanostars, it was difficult to identify single nanostars, demonstrating efficient intracellular uptake in vivo upon intra-tumoral injection.

## 4. Discussion

We report the use of gold nanostars as theranostic agent againsts cancer using their photoacoustic and CT imaging capabilities to guide PTT after intratumoral injection. This strategy has clinical potential for the ablation of superficially localized tumor masses, or for aiming at complete tumor cell eradication during tumor resection surgery. The clinical potential for a similar approach was demonstrated before, using magnetic nanoparticles [31,46].

Hereby, the efficient cellular uptake of nanostars into tumor cells is important for later in vivo applications. A first criterion is the size where two processes need to be considered: (1) optimal NP/cell interaction and (2) optimal tumor retention [47,48]. Nanoparticles having a hydrodynamic diameter above 100 nm are shown to have better tumor retention after intra-tumoral injection compared to smaller sized particles. In vitro studies, on the other hand, have shown that nanoparticles of around 50 nm diameter have the ideal size for cell interaction, while the cell uptake is less efficient for larger nanoparticles [47]. We have optimized nanostars to have a hydrodynamic diameter of around 100 nm in serum, which is the cutoff-value for optimal tumor retention and efficient tumor cell interaction. Second, the protrusions of the nanostars with a small curvature potentially increase cellular uptake by increasing their membrane interaction [48]. Third, the anionic particles used in this and previous studies show a much higher stability compared to previously described cationic particles and still result in high cellular accumulation due to electrostatic interactions with the cell membrane [47]. Consequently, we were able to show that nanostars were taken up reaching a gold concentration 11.28 ± 0.82 pg Au/cell after 24 h incubation, indicating that they are suitable for efficient passive labeling of tumor cells.

Given their favorable properties for tumor cell uptake, we demonstrated that nanostars can be used as an in vitro and in vivo theranostic agent combining both dual-modality imaging (PAI and CT) and PTT. Pure nanostar suspensions resulted in an almost equally high CNR for both imaging modalities. This is different for in vitro cell experiments, where a higher CNR for PAI is measured compared to CT. This is due to the lower background signal of PAI compared to CT. Effective PTT was possible for nanostar labeled tumor cells in vitro. In contrast, unlabeled cells remained unaffected by laser treatment. For comparing the PTT effectiveness of different nanoparticles, the irradiation power that is needed to cause cell death is used as an indicator. The radiation power of 7 W/cm^2^ used in our study is in the same range or lower as in similar experiments that were performed with nanorods (10 W/cm^2^) [49], nanoshells (35 W/cm^2^) [50], and nanocages (5 W/cm^2^) [51]. The relatively low radiation power used for our nanostars indicates their efficiency when compared with many nanostars reported in the literature (15 W/cm^2^, 38 W/cm^2^ and 0.2 W/cm^2^) [13,19,21]. The maximum absorption of the nanostars used in our study is just outside the preferred window of 750–900 nm. Here, we had to find a compromise between the reduced size to enable possible delivery through leaky blood vessels and tumor cell uptake on one side and a red-shifted absorption band which would have resulted in larger nanostars [39]. In addition to inherent characteristics of the nanoparticles (absorption coefficient, size, and wavelength), other factors like cellular labeling conditions, chemical coatings, biofunctionalization of the AuNPs, cellular properties, and location of the AuNPs in the cells are equally important when comparing AuNPs regarding the irradiation power for PTT [35,48,52,53]. Consequently, there is a need for more in-depth and standardized studies for comparing different shapes of AuNPs and their theranostic potential as well as the stability of coating material at temperatures of 37 °C and above [35]. Although, we did not see any adverse effects on cell biology after incubation of cells with PEGylated Au-nanostars in a previous study [22], it cannot be excluded that the non-covalent Au-S-bond shows instability at elevated temperatures [35].

In addition to in vitro experiments, we evaluated the use of in vivo imaging modalities (CT and PAI) to monitor intra-tumor nanostar delivery and their corresponding contrast generating capabilities. With nanostars having optimal dimensions for intra-tumoral delivery, efficient nanostar accumulation in the tumor 24 h after injection was confirmed by PAI and CT in vivo and ex vivo TEM. These nanostars were densely packed in endosomes, as visualized by TEM. Concerning the contrast generation of these nanostars in vivo, for PAI a significantly higher CNR (approx. 80) was observed compared to CT (approx. 25). The high sensitivity of PAI could potentially be further improved by coating the nanostars with a silica shell [54]. An additional advantage of PAI over CT is its high temporal resolution, while CT provides additional anatomical information. We were able to demonstrate that both imaging techniques can be used for intra-tumoral nanostar detection if high local concentrations can be achieved. By combining CT and PAI in a dual-modality approach using highly sensitive gold nanostars, PAI could provide rapid and detailed information on local nanostar distribution and their temporal changes, while CT could provide a full body scan with more detailed anatomical information. Compared to dual contrast agents that use radio-nuclei labeling like for PET-MRI [55,56], the combination of CT and PAI provides long-lasting contrast.

We have also confirmed the efficiency of the nanostars for PTT. Due to their star shape, they have a high absorption coefficient resulting in a large temperature increase of Δ25 °C when irradiated with a laser power of only 2 W/cm^2^. Reaching a local temperature higher than 43 °C will result in necrosis of tumor cells [28,57]. In vivo necrosis was confirmed in this study after tumor irradiation (2 W/cm^2^), while the control tumors did not show any side effects of laser irradiation. Using laser powers higher than the prescribed laser norm of 0.2 W/cm^2^ defined by the American Laser Institute did not damage tissue when irradiating the control sample as confirmed by histology (Figure 7) [58]. Compared to nanoshells (4 W/cm^2^) [50] and nanorods (2 W/cm^2^) [30], the nanostars require the same magnitude of laser power to induce ablation after intratumoral injection. However, comparisons between different nanoparticles to assess their therapeutic efficiency are difficult due to the different experimental conditions used and incomplete or limited information available.

The in vivo efficiency of PTT was shown in detail using BLI, MRI, and histology (Figure 6). The BLI signal intensity decrease one day after irradiation was either caused by a decrease in tumor cell viability, destroyed tissue, vessel patency or a combination of them. As a significant decrease in tumor volume is noticed by MRI from day 8 onwards, the decrease in BLI signal is most likely caused by tumor cell death. The large number of necrotic cells seen in H&E staining of the nanostar-injected tumors confirmed this hypothesis. Regrowth of the tumor after PTT treatment was observed by BLI after day 8. This is most likely due to an incomplete delivery of gold nanostars to all tumor cells so that some residual tumor tissue/cells remain as also confirmed by MRI and histology after 15 days. As local injections of nanostars will result in an inhomogeneous distribution within the tumor, applying multiple injections and irradiations will most likely improve outcome [23]. Hereby, the combination with imaging techniques that provide information on the nanostar distribution and therapy outcome is of utmost importance. Additional treatment options to improve outcome are a combination of PTT with chemotherapy, photodynamic therapy, or the introduction of tumor-targeting moieties to the nanostars [59,60].

## 5. Conclusions

We have demonstrated an optimized synthesis and application of gold nanostars as a theranostic agent for combined multimodal imaging using CT, PAI and photothermal therapy. Due to the red shift of the LSPR band and the high absorption coefficient, these nanostars could have a better theranostic outcome compared to other shaped nanoparticles. The gold nanostars created significant contrast in both CT and PAI and proved to be an effective therapeutic agent for PTT, due to a successful passive uptake by the tumor cells. Future work should focus on the intravenous delivery of gold nanostars (passively or actively) leading most likely to further improvements in the nanotheranostics field, aiming for imaging and therapy of specific tumor types, which also has the potential to target and treat tumor metastases.

## Supplementary Materials

The following are available online at https://www.mdpi.com/2079-4991/10/11/2112/s1, Figure S1: UV-Vis absorption spectroscopy of nanostars. Figure S2: UV-Vis absorption spectroscopy, DLS intensity plots and zeta-potential measurements of nanostars. Figure S3: Intracellular gold concentration using ICP-OES. Figure S4: Temperature profile, fluorescence and bright field microscopy of gold nanostars and irradiated tumor tissue. Figure S5: In vivo BLI before and after nanostar injection. Figure S6: Bright field microscopy images and histology of control and nanostar-injected tumors.
