# Supplementary material for: Assessment of the Theranostic Potential of Gold Nanostars—A Multimodal Imaging and Photothermal Treatment Study
Source: Nanomaterials (Basel). 2020 Oct 23;10(11):2112. doi: 10.3390/nano10112112 (PMC7690792; doi:10.3390/nano10112112)
Supplement: Supplementary file 1 [file nanomaterials-10-02112-s001.pdf]

nanomaterials

MDPI

Supplementary Material

Assessment of the Theranostic Potential of Gold Nanostars—A Multimodal Imaging and Photothermal Treatment Study

Antoine D'Hollander [1,2,3], Greetje Vande Velde [1,2], Hilde Jans [3], Bram Vanspauwen [3], Elien Vermeersch [1,2], Jithin Jose [4], Tom Struys [5], Tim Stakenborg [3], Liesbet Lagae [3,6] and Uwe Himmelreich [1,2,*]

[1] Biomedical MRI, Department of Imaging and Pathology, Faculty of Medicine, University of Leuven, Herestraat 49, 3000 Leuven, Belgium; antoinedhollander@hotmail.com (A.D.); greetje.vandevelde@kuleuven.be (G.V.V.); elien.vermeersch@kuleuven.be (E.V.)

[2] Molecular Small Animal Imaging Center (MoSAIC), University of Leuven, Herestraat 49, 3000 Leuven, Belgium

[3] Department of Life Science Technology, IMEC, Kapeldreef 75, 3001 Leuven, Belgium; Hilde.Jans@imec.be (H.J.); brmvanspauwen@gmail.com (B.V.); Tim.Stakenborg@imec.be (T.S.); liesbet.lagae@imec.be (L.L.)

[4] Fujifilm Visualsonics, Joop Geesinkweg140, 1114 AB Amsterdam, The Netherlands; jithin.jose@fujifilm.com

[5] Lab of Histology, Biomedical Research Institute, Hasselt University, Agora Laan Gebouw C, 3590 Diepenbeek, Belgium; tom.struys@sprofit.com

[6] Department of Physics, Faculty of Sciences, Laboratory of Soft Matter and Biophysics, University of Leuven, Celestijnenlaan 200D, 3001 Leuven, Belgium

* Correspondence: uwe.himmelreich@kuleuven.be; Tel.: +32-16-330925

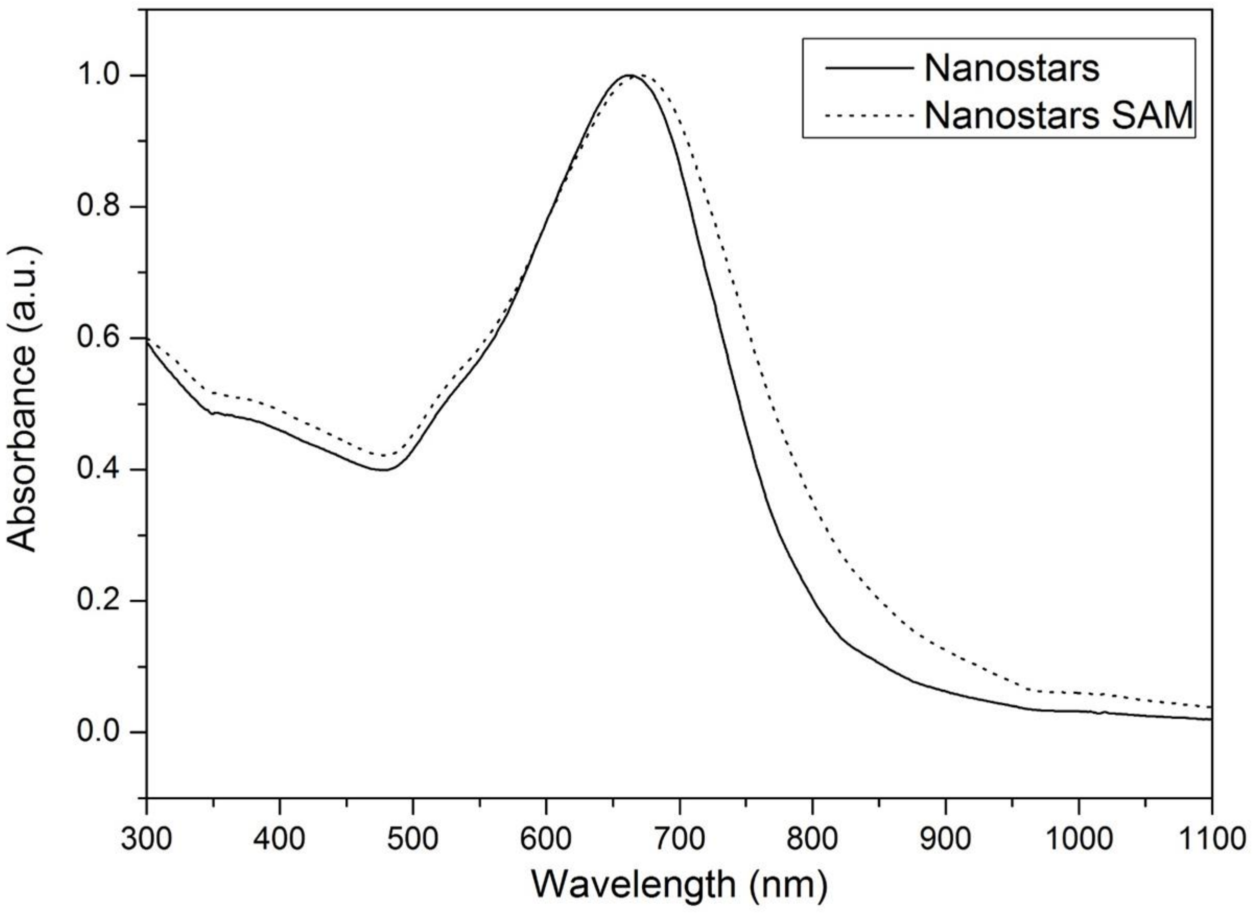

Figure S1. UV-Vis absorption spectroscopy of nanostars, normalized to absorbance 1, without and with SAM functionalization. The red-shift of the plasmon band (9 nm) was due to a change in the refractive index.

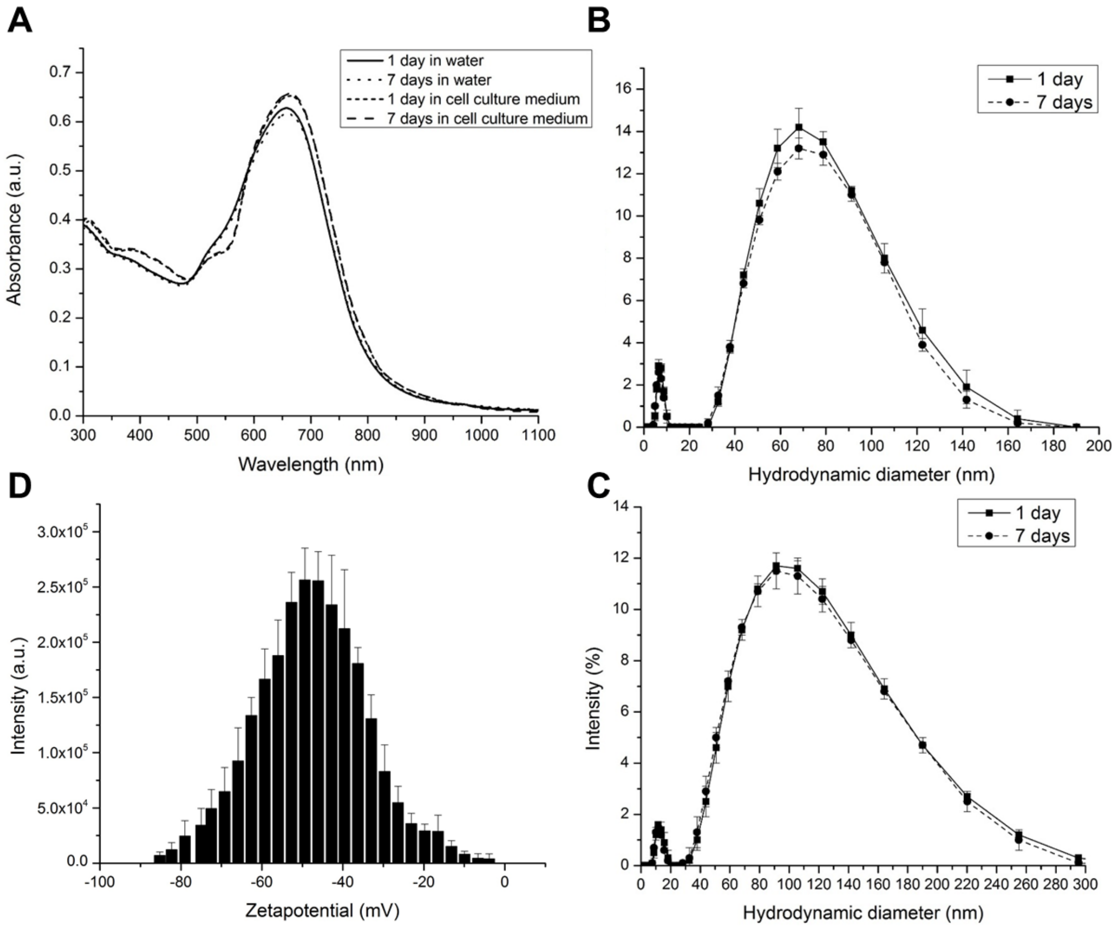

Figure S2. A. UV-Vis absorption spectroscopy of nanostars for 1 and 7 days in either water or cell culture medium. No shift of band broadening was observed indicating that the nanostars are stable in water and cell culture medium B. DLS intensity plots of the nanostars in water for 1 and 7 days showed no increase or decrease in diameter. C. DLS intensity plots of the nanostars in cell culture medium for 1 and 7 days. A difference in diameter is observed compared to the nanostars in water (increase by 25.63 ± 1.23 nm), indicating the formation of a protein corona around the nanostars. Still after 7 days, there was no further increase observed compared to the time point at 1 day, confirming the nanostars' stability in biological medium. D. The zeta-potential of the anionic nanostars in water with an average value of -41.3 ± 1.2 mV.

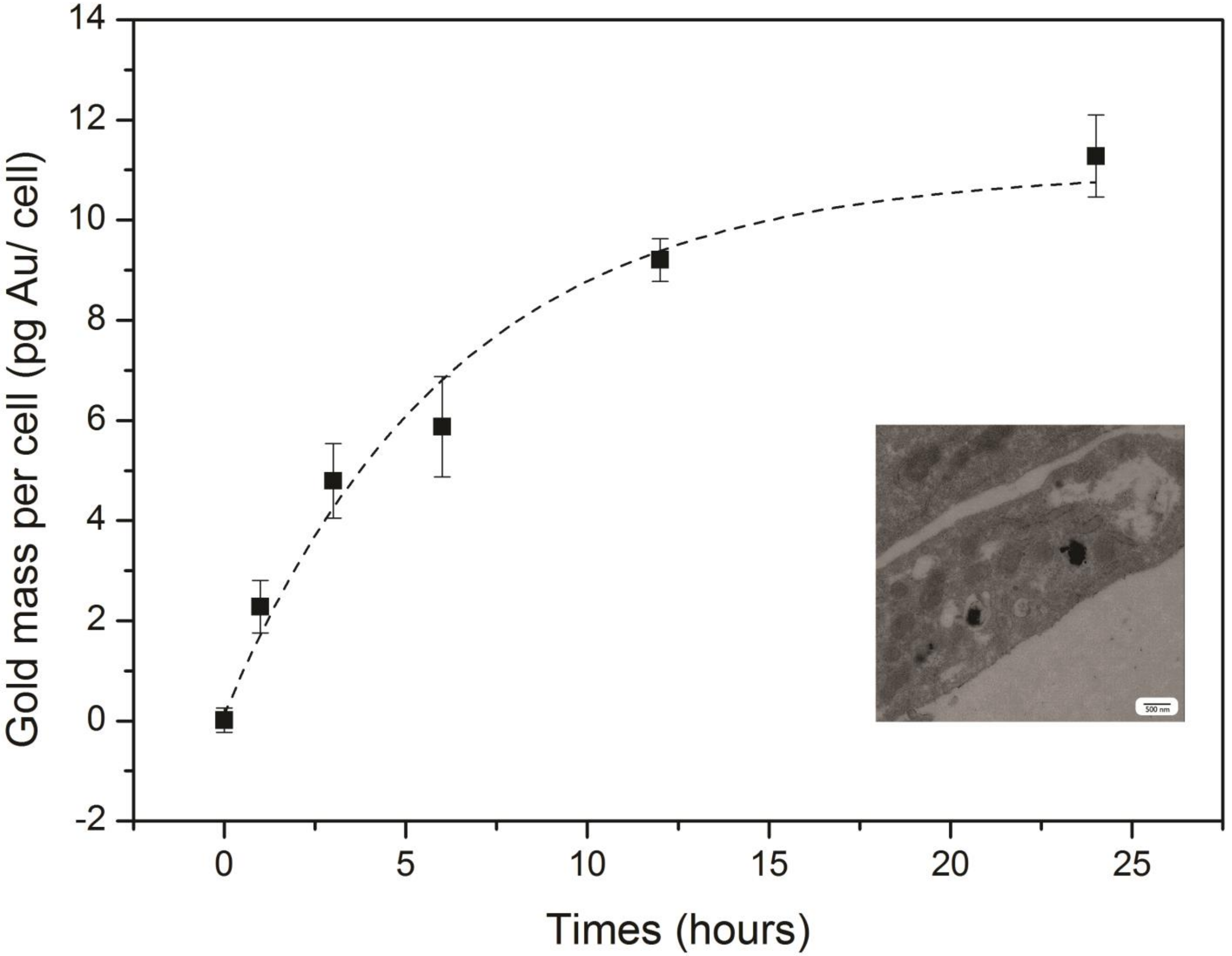

Figure S3. Intracellular gold concentration of different incubation times determined by ICP-OES. As inset, TEM images visualizing the nanostars inside the tumor cells.

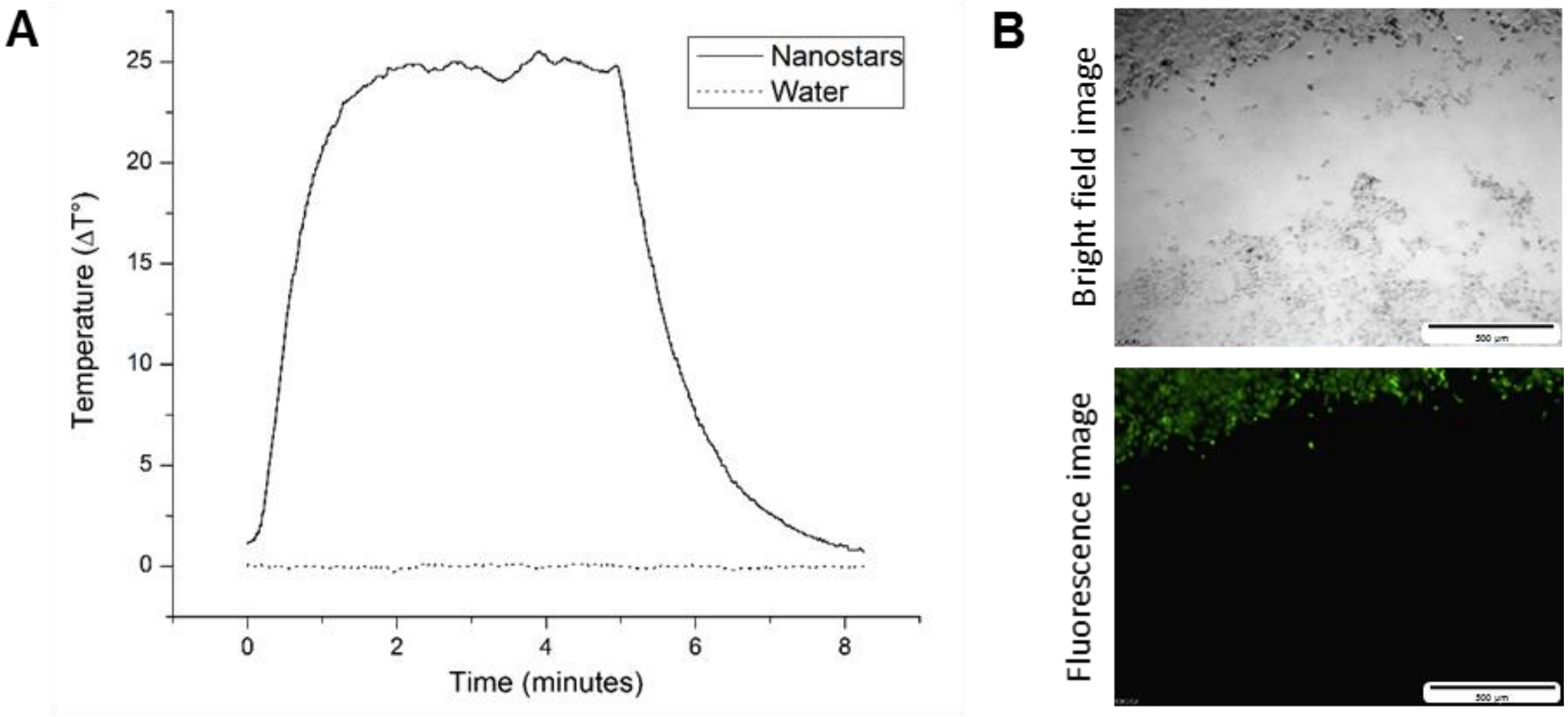

Figure S4. A. Temperature profile of tubes that were filled with either gold nanostars or water and were irradiated with a continuous laser. B. Fluorescence (a) and bright field microscopy (b) images of irradiated tumor tissue at the border region of the laser spot. A clear border is visible in the calcein AM stained images, making distinction between life and dead cells possible.

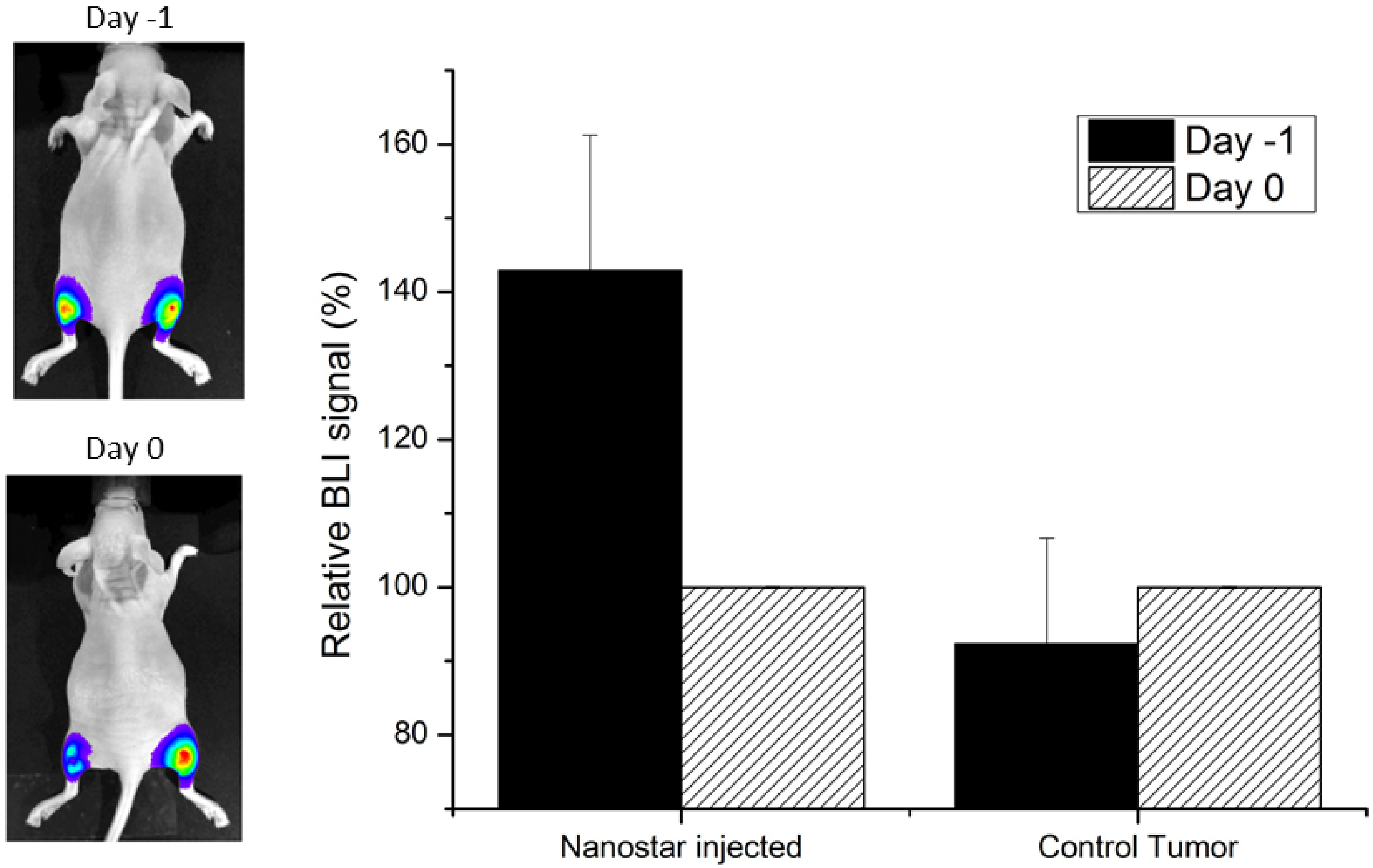

Figure S5. *In vivo* BLI before nanostar (day -1) before after nanostar (day 0) injected illustrated in a color-coded intensity map on the mice. A quantification of the BLI signal intensity relative to day 0 (set 100%) is plotted for both nanostar-injected and control (PBS-injected) tumors (RT) (n=6, tumors per condition), where a decrease is shown after nanostar injection.

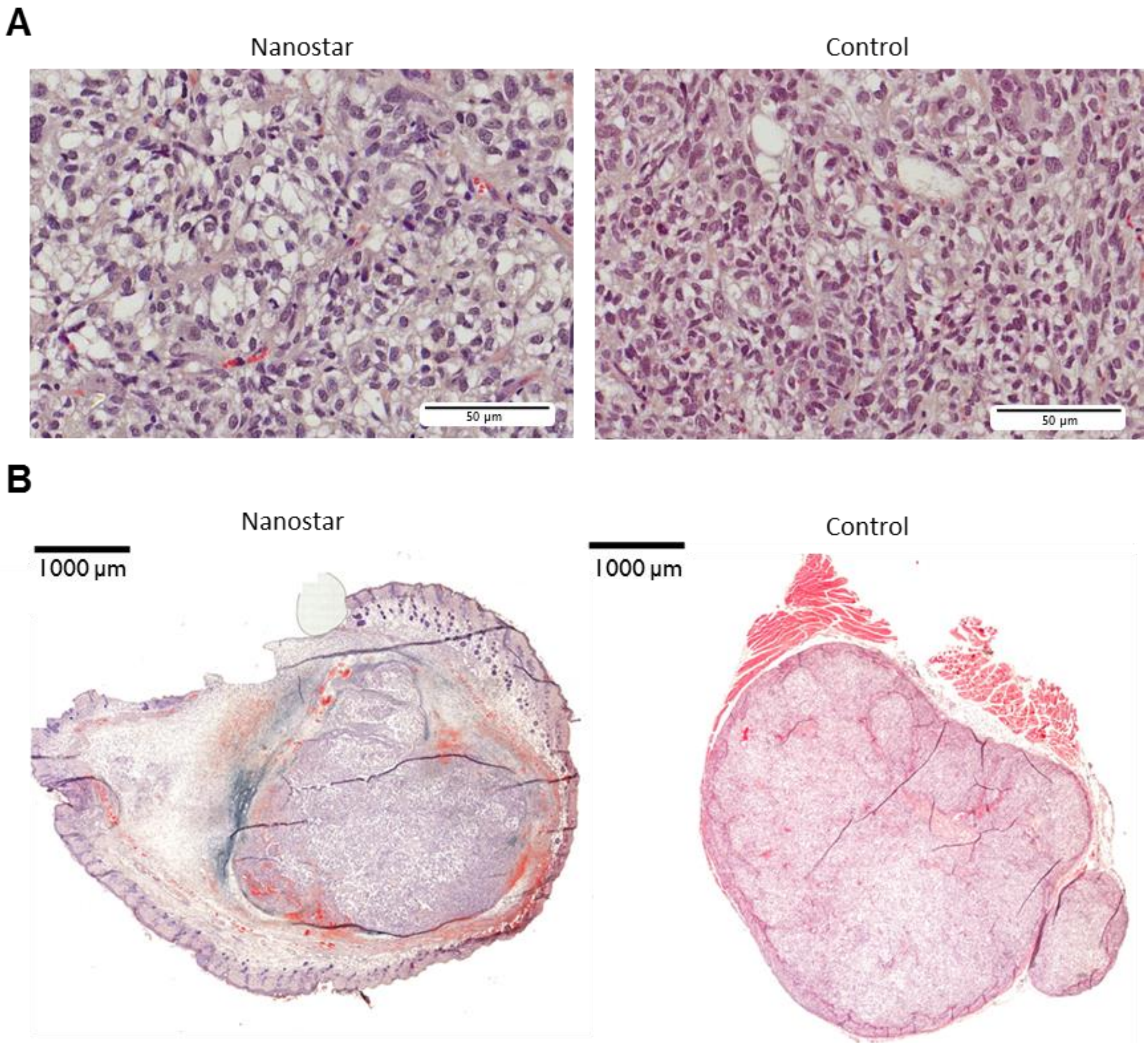

Figure S6. A. Bright field microscopy images of control (right) and nanostar (left) injected H&E stained tumor sections, where the animals are sacrificed 15 days after therapy. No necrotic cells are visible in the tumor sections indicating the regrowth of the tumors. B. A complete overview of a histological section of either the tumor injected with nanostars (left) or PBS (right) after therapy. The blue 'cloud' in the tumor is caused by the gold nanostars, which is absent in the control tumor.
